# Shot Characteristics Based on Match Period in Elite Table Tennis Matches

**DOI:** 10.3389/fpsyg.2021.745546

**Published:** 2021-10-18

**Authors:** Jie Wang

**Affiliations:** Key Laboratory of Diagnosis and Analysis of Skills and Tactics in Sports, College of Physical Education and Sports Training, Shanghai University of Sport, Shanghai, China

**Keywords:** table tennis, set point, performance analysis, contextual-related variable, notational analysis

## Abstract

This study aimed to compare the shot characteristics amongst different match periods in table tennis matches. For the shot characteristics of position, type, placement and efficacy, 13 men’s singles matches comprising 72 sets from a round of 16 to the final in the 2019 World Table Tennis Championships were selected for notational analysis. Kruskal–Wallis and Mann–Whitney tests were conducted to quantify the differences in various categories of each variable amongst the initial, intermediate and ending match periods. There are no significant differences in each distribution between initial and intermediate periods. From intermediate to ending periods, significant decreases were found in chop type (*p* < 0.01) and middle long placement (*p* < 0.01), whereas a significant increase was found in backhand half placement (*p* < 0.05). By comparing the initial and ending periods, significant decreases were found in middle backhand turn position (*p* < 0.05), middle long placement (*p* < 0.01), chop (*p* < 0.01), and poor shots (*p* < 0.05). Therefore, shot characteristics in table tennis matches are match period related, and the main differences existed between the beginning and the ending period. From initial to ending period, the frequency of applying the middle long placements is decreasing. At the end of every set, the players adopted a safe stroke position, performed offensive stroke types and deployed flexible stroke placement. Results suggested that coaches can establish different scenarios on the basis of varying match periods for players’ training.

## Introduction

Sport performance is influenced by multiple factors, such as the task at hand, the environment and players’ respective states (Newell, 1986; Kolman et al., 2019). To achieve optimal performance, athletes should maximize their own advantages and provide the proper responses to special situations. In table tennis, the determinants of players’ performances are multidimensional, such as good physical fitness (Zagatto et al., 2010; Malagoli Lanzoni et al., 2014), psychological control and technical and tactical skills (Munivrana et al., 2015). Given the structural complexities of technical and tactical skills, these abilities are the most important determinants compared with other factors (Malagoli Lanzoni et al., 2014; Munivrana et al., 2015). Notational analysis is an effective method to evaluate the technical–tactical actions in racket sports (Hughes and Franks, 2004; Malagoli Lanzoni et al., 2014). Performance indicators play an important role in notational analysis because they relate to successful outcomes (Hughes and Bartlett, 2002). In table tennis competitions, the descriptors of shot characteristics are used most frequently (Malagoli Lanzoni et al., 2014).

The indicators of shot characteristics in table tennis include stroke type (Yu et al., 2007; Zhang et al., 2010; Loh and Krasilshchikov, 2015; Wang, 2019), stroke position (Yu et al., 2008; Wang, 2019), stroke efficacy (Malagoli Lanzoni and Lobietti, 2008; Yu et al., 2008; Malagoli Lanzoni et al., 2010; Zhang et al., 2010; Wang, 2019) and the impact position of the ball on the table (Yu et al., 2008; Baca and Kornfeind, 2009; Zhang et al., 2010; Malagoli Lanzoni et al., 2011b; Wang, 2019). Shot characteristics in high-level table tennis matches have attracted considerable interest from analysts and scholars. However, the majority of these studies have only focused on one or more aspects of shot characteristics.

Performance analysis revealed that game performance is time- and context-dependent, which occurs in a dynamic and interactive manner (McGarry et al., 2002; Glazier, 2010; Marcelino et al., 2011). Similar to other net sports, the behavior of table tennis players has emerged in a dynamic manner on the basis of different situations (Glazier, 2010; Gómez et al., 2017) and is influenced by contextual variables (e.g., game duration, biggest led, quality of opposition) (Malagoli Lanzoni et al., 2011a; Marcelino et al., 2012; Gómez et al., 2017). The analysis of contextual variables is conducive to gaining insights into performances in specific situations and obtaining substantial objective understanding of shot characteristics (Marcelino et al., 2012; Gómez et al., 2017). Given the importance of contextual-related factors, one of the important tasks of analysts is to determine additional potential contextual-related variables, clarify their influences on game performance and serve for training and competition.

Amongst various contextual-related factors, quality of opposition (Gómez et al., 2017) and phase of competition (Leite et al., 2017) are important factors in table tennis. However, these studies have merely focused on part of players’ performances influenced by contextual factors, such as the influence of the period of competition on temporal structure and contextual-related variables on serve and receiving performance. A few of these studies have extensively studied shot characteristics, which is the most important characteristics of table tennis players.

Match period, which is a time- and contextual-related factor, plays an important role in elite volleyball matches. A study has indicated considerable differences in attack and serve performances during different match periods (Marcelino et al., 2012). Table tennis analysts are also concerned with the possible performance differences based on various match periods and use such differences to complete match analysis (Fuchs et al., 2018). Most of these studies have divided match periods into three parts: beginning, middle and crucial parts of a set (An, 2007; Yu et al., 2009; Deng, 2018; Fuchs et al., 2018). In view of tactics performance, An (2007) followed a three-phase method (Wu and Li, 1992), analyzed high-level men table tennis players and found no significant difference in various periods. In view of techniques and tactics performance, Deng (2018) studied elite women table tennis players and found substantial differences in the beginning, middle and crucial parts of a set. Given the use of different performance indices, no consistent conclusions have been drawn on the match period’s influence on elite players’ performance. Moreover, given that players in table tennis matches will experience situations as adaption, confrontation and run-off from beginning to the end of every set (An, 2007), they must use corresponding actions to manage different situations. Based on previous studies, the assumption is that shot characteristics in high-level table tennis competitions vary on the basis of different match periods. The present study aims to identify shot characteristics regarding match periods (i.e., initial period, intermediate period, and ending period) during each set in high-level table tennis matches. This objective will deepen our understanding of performance analysis in table tennis, thereby benefitting coaches’ decision making related to training and competition.

## Materials and Methods

### Sample

A total of 13 men’s single competitions from a round of 16 to the final in the 2019 World Table Tennis Championships were selected, with 72 sets corresponding to 144 player-sets. There was a total of 6,392 videos included for analysis. Owing to some missing video clips, one video of rallies amongst these matches were excluded in the analyses. The videos were downloaded from bilibili.com. This study was approved by the College of Physical Education and Sports Training Academic Committee at Shanghai University of Sport.

### Data Collection

A system observational tool was developed for data recording and analysis, in which various attributes of every player’s stroke in the competition were collected. Serve, which is a type of unique shot and not to be forced by the opponent, was excluded in the analysis. For videos with unclear images, plays were judged using best knowledge. Two observers were recruited to analyze and record data. One has over 15 years of experience in table tennis analysis and research. The other is a table tennis player qualified by the Chinese Table Tennis Association as Level A to test for inter-rater reliabilities. Both of them were trained to get familiar with the process of observation and understand the meaning of each observation index. One competition was selected randomly and watched by two observers independently. The data was collected to test inter-observer reliability. Then, the competition was watched again by one observer after 1 week to test the intra-observer reliability. Kappa values were calculated on the basis of intergroup measurement data and intragroup repeated measurement data to characterize inter- and intra-observer reliabilities. For stroke type, stroke efficacy, stroke position and ball placement of stroke, the values of kappa were greater than or equal to 0.88, for intra-observer reliabilities; greater than or equal to 0.85, for inter-observer reliabilities.

### Observation Parameters

Stroke type is the most important stroke feature, which can be evaluated by the direction of the shot, spin and arc of the ball. Following the related literature (Zhang et al., 2010; Malagoli Lanzoni et al., 2013, 2014; Wang, 2019), stroke type was divided into topspin, smash, flip, chop, chopping short, block, and others.

Players should constantly adjust their positions in the game to choose the most suitable form to generate force. Stroke position is the position of players returning a coming ball and includes four types: forehand, backhand, pivot and backhand turn. The classification mode is based on the literature (Yu et al., 2008; Wang, 2019). The definition of stroke position is consistent with Wang’s (2019) definition.

The classification of stroke efficacy (i.e., very good, good, neutral, poor, very poor) is in accordance with previous research (Malagoli Lanzoni et al., 2011a) and its definition followed Wang’s (2019) definition. A very good stroke leads into a point or obvious initiative of a player. A good stroke leads into a certain extent initiative of a player. A neutral stroke lead into a balance between the player and the fighter. A poor stroke leads into the fighter’s initiative. A very poor stroke leads into the point or obvious initiative of the fighter. Stroke efficacy is the embodiment of comprehensive application of technology and tactics of players.

Stroke placement is the landing area of a ball when hit by a table tennis player. Based on related studies (Zhang et al., 2010; Malagoli Lanzoni et al., 2014), the stroke placement is classified into nine different parts: forehand short, forehand half, forehand long, middle short, middle half, middle long, backhand short, backhand half and backhand long placements. The definitions followed Wang’s (2019) research.

Match periods, which are the group variable in the current study, are the divisions in a set of a game in chronological order, and the definition of match periods follow a previous study (Deng, 2018). Based on the purpose of the study, match periods are divided into three periods, namely, the initial, intermediate and ending periods in a set of a game. The divisions depend on the points on both sides of a set. The game is in the initial periods when the score is not more than 4 points on both sides. The game is in the intermediate period when either of the two sides in the set is more than 4 points and the score of both sides is not more than 8 points. If one side in the set scores more than 8 points, then the game proceeds to the ending period.

### Statistical Analysis

Because the study aimed to examine the differences in the four dependent variables (i.e., stroke position, stroke type, stroke placement, and stroke efficacy) by game periods (i.e., initial period, intermediate period, and ending period), which is the group variable used for the data analysis, descriptive analysis (i.e., means, and standard deviations) was conducted first. Then Shapiro–Wilk test was performed to test whether the assumption of normality was met for ANOVA. Because the data were not normally distributed (i.e., Shapiro–Wilk test was significant, *p* < 0.05), one-way ANOVA was not utilized in the current study. Instead, Kruskal–Wallis tests were performed because it does not require the data to be normally distributed. The dependent variables were stroke position, stroke type, stroke placement, and stroke efficacy while the three groups were game periods (i.e., initial period, intermediate period and ending period). Moreover, Benjamini-Hochberg correction was used to further identify differences between multiple groups. The reason for applying Benjamini-Hochberg correction is to decrease the false discovery rate to control for the fact that sometimes small *p*-values (i.e., less than 5%) happen by chance when multiple groups were compared (Benjamini and Hochberg, 1995). The *post hoc* tests (i.e., Pairwise Mann–Whitney tests) were performed to identify the differences among groups for significant Kruskal-Willis tests. Statistical significance was set at *p* < 0.05. All statistical analyses were performed using SPSS 24.0.

## Results

The distributions of each category related to shot characteristics in the initial, intermediate and ending periods are shown in Table 1, indicating no significant differences of each distribution between the initial and intermediate periods. Significant differences existed in the stroke position, stroke type, stroke placements and stroke efficacy between the initial and ending periods. Between the intermediate and ending periods, considerable significant differences were found in stroke type and stroke placement.

**TABLE 1:** Mean ± standard deviation of percentages of stroke position, stroke type, stroke placement, stroke efficacy, and the differences for each category among different periods.

		**Initial period (*n* = 144)**	**Intermediate period (*n* = 144)**	**Ending period (*n* = 144)**
Stroke position	Forehand (%)	29.79 ± 13.68	30.23 ± 14.62	31.29 ± 22.91
	Backhand (%)	38.41 ± 15.76	39.34 ± 16.62	39.04 ± 22.65
	Pivot (%)	20.50 ± 14.94	20.93 ± 15.93	19.36 ± 21.61
	Backhand turn (%)	11.29 ± 12.01	9.51 ± 12.11	9.62 ± 14.32*
Stroke type	Topspin (%)	57.45 ± 16.21	59.96 ± 15.18	54.84 ± 23.66
	Smash (%)	0.34 ± 1.97	0.29 ± 1.61	0.23 ± 1.69
	Flip (%)	7.98 ± 9.55	7.92 ± 9.81	7.71 ± 12.50
	Chop (%)	6.44 ± 7.17	6.83 ± 8.79	4.37 ± 8.15*#
	Chopping short (%)	12.33 ± 9.48	12.07 ± 9.75	13.73 ± 14.45
	Block (%)	9.26 ± 9.94	7.65 ± 8.96	11.70 ± 16.92
	Others (%)	6.20 ± 9.95	5.28 ± 8.23	6.72 ± 12.73
Stroke placement	Forehand short (%)	2.27 ± 5.07	3.09 ± 6.21	4.80 ± 15.04
	Forehand half (%)	3.45 ± 5.54	4.06 ± 7.63	5.24 ± 14.84
	Forehand long (%)	17.66 ± 12.95	16.62 ± 14.40	17.14 ± 20.77
	Middle short (%)	4.77 ± 7.84	4.10 ± 6.62	4.87 ± 13.16
	Middle half (%)	10.03 ± 11.06	8.91 ± 13.28	11.75 ± 17.50
	Middle long (%)	20.5 ± 15.84	20.35 ± 15.67	16.62 ± 21.09*#
	Backhand short (%)	0.73 ± 3.21	0.65 ± 2.94	0.69 ± 4.24
	Backhand half (%)	3.23 ± 5.56	4.45 ± 7.70	3.43 ± 8.27#
	Backhand long (%)	37.35 ± 19.98	37.78 ± 19.52	34.07 ± 27.12
Stroke efficacy	Very good (%)	9.94 ± 9.75	9.12 ± 10.43	9.21 ± 13.90
	Good (%)	20.03 ± 18.49	20.30 ± 18.81	21.23 ± 25.73
	Neutral (%)	32.29 ± 22.04	35.17 ± 22.96	31.33 ± 26.35
	Poor (%)	12.34 ± 12.30	11.54 ± 13.26	12.09 ± 17.80*
	Very poor (%)	26.41 ± 12.62	23.88 ± 12.90	26.15 ± 20.00

*Different from initial period; ^#^different from intermediate period.

For the distribution of stroke position, the proportion of backhand turn significantly decreased (*p* < 0.05) from 11.29 to 9.62% from the initial to ending periods. For the distribution of stroke type, the proportion of chop significantly decreased (*p* < 0.01) from 6.83 to 4.37% from the intermediate to ending periods. Significant differences (*p* < 0.01, from 6.44 to 4.17%) existed between the initial and intermediate periods as well. For the distribution of stroke placement, the proportions of the middle long and backhand half have shown substantial differences. In terms of the placement of middle long, considerable difference was found between the initial and ending periods (*p* < 0.01) and between the intermediate and ending periods (*p* < 0.01). Overall, a downward trend of middle long placement has been shown from the initial to ending periods, in which the values are 20.5, 20.35, and 16.62%, respectively. Given the placement of the backhand half, significant differences were found between the intermediate and ending periods (*p* < 0.05). From the intermediate to the end of a set, the proportion backhand half placement decreased, in which the values are 4.45 and 3.43%, respectively. For the distribution of stroke efficacy, poor strokes significantly decreased (*p* < 0.05) from 12.34 to 12.09% between the initial and ending periods.

## Discussion

This study aimed to evaluate the differences of stroke characteristics of elite table tennis players in different match periods. The results indicated no differences between the initial and intermediate periods, although some significant differences existed between the initial and ending periods and between the intermediate and ending periods.

Table tennis matches often undergo three stages, namely, adaptation, confrontation and decisive stages (An, 2007). At the beginning of the game, both sides of the game are often in the adaptive state, in the middle of the game, the two sides are in the antagonistic state and at the end of the game, both sides of the game are in the decisive stage. With changes in the game situation, players may use the corresponding techniques and tactics to adapt to the changing situation and show different shot characteristics. Relations exist between the profiles of skills and tactics of players and match periods in volleyball matches (Marcelino et al., 2012). However, no consistent conclusions have been reached on whether the shot characteristics of elite table tennis players were related to the game periods. Some studies have suggested a relationship between the player’s short characteristics and game periods (Deng, 2018), although some did not (An, 2007). Our findings supported the former’s conclusions and suggested a relationship between them. Our results indicated that significant differences existed in all dimensions of shot characteristics: stroke position, stroke type, stroke placement and stroke efficacy.

### Differences in Stroke Position

Stroke position is the position of a player when hitting the ball. A suitable position selection is beneficial to athletes to deliver strength and perform the corresponding skills. The results of the current study indicated that the distribution of the stroke position of table tennis players is similar between intermediate and ending match periods. A significant decrease is found in the proportion of backhand turn between initial and ending period. The backswing time of backhand turn stroke is relatively short, and the difficulty of execution is relatively large. Generally, the strokes of backhand turn act as a tactical arrangement. The decrease of the usage ratio of backhand turn indicates that most players may choose a more secure position to execute skills at the end of a competition. At the end of the game, the athletes will become more conservative (Ren et al., 2013). They will be more inclined to execute technology and tactics in a conventional way. This result suggests that the athlete’s stroke position is closely related to the athlete’s psychology. High-level athletes should pay special attention to psychological training in a particular situation.

### Differences in Stroke Type

Stroke type, which is the comprehensive embodiment of players’ speed, power and making spin, is the most important stroke feature in table tennis. Some shots are used for attack (e.g., topspin, smash and flip), defense (e.g., block) and control (e.g., chop and chopping short). The preceding research results indicated that from the initial to ending periods, the ratio of the stroke types of offensive and defensive did not change, whilst the percentage of the control type (chop) decreased. The changes may be caused by psychological changes in both sides. At the beginning of the games, players from both sides are in the adaptation period and use different techniques to test each other (Shi et al., 2015). The proportion of controlling technology (chop) is relatively large. At the end of a game, the side with the lead attempts to finish the game as soon as possible by scoring on offense, and the backward side strives to attack to gain the last chance (An, 2007), the techniques are mainly switched between offense and defense and the game goes to decisive stages. Therefore, offensive and defensive techniques are preferred by both sides of the game, the use of control techniques will decline as they merely act as a transition technique at the end of a game. In terms of the reliability of technical execution, chop will produce long placement, which is more likely to be caught by the opponent. Therefore, the use of chop could be reduced in the final stage of a game. The changes of stroke types can also reflect those athletes are more conservative in the final stage of the game. The results suggest that despite the pressure on both sides in the final stages of the game, the players should remain calm. As an important transitional shot type, the use of chop techniques can reduce the direct error and get well prepared for the next shot. Topspin is still the most important way to hit the ball. In daily training, we should pay attention to the practice of topspin.

### Differences in Stroke Placement

Stroke placement, which is the landing area of the ball, is an important tactical aspect in table tennis (Malagoli Lanzoni et al., 2011a). Depending on the distance from the net, placements are divided into short, middle and long. Generally, catching a long placement is easy for the opposite side. If the ratio of long placements decreases, then the competition is considerably difficult. The results of this study indicated that from the initial to ending periods of a game, the ratio of the middle long placement decreases and the ratio of forehand short placement increases significantly. Undoubtedly, the difficulty of the game increased from the initial to ending periods. Both sides of the game want to restrict each other by increasing the difficulty of placement. Generally, players more easily execute the forehand placement than the middle and backhand. When faced with the choice of stroke placements under time pressure, players tend to switch from backhand to forehand rather than from backhand to forehand. However, at the end of the game, to increase the difficulty of the opponent and facilitate their own implementation, the best choice of players is to perform backhand placements. This tactic led to an increase in the use of the backhand half and a decrease in the use of the middle long. In fact, the bigger change in the stroke placement is the equalization of landing area of ball. At the beginning of a game, most of landing areas are in the areas far away from the net, however, at the end of a game, the landing area has changed. The proportion of long ball placement decreased, and the proportion of short and half-court ball placement has increased. The players are more flexible in their deployment of ball placement at the ending periods (Ding and Ling, 2013). The change of landing point is important tactical implementation methods of athletes in table tennis. The change of placement is to disrupt the rhythm of the opponent. Excellent athletes are especially good at changing their placement in the final stage of the game. Coaches should cultivate the ability of athletes to cope with the change of placement in daily training.

### Differences in Stroke Efficacy

Stroke efficacy reflects the effect of the comprehensive application of technology, tactics and psychological adjustment of players. From the initial to the end periods of the game, the percentage of poor shots decreases significantly. The percentages of particularly good and good shots increase. Given that the psychological pressure faced by athletes in the initial stages of a game is relatively low, more shots are optional, and the proportion of poor shots are relatively large. At the end of a game, pressure mounts on both sides, and the leading side tends to take advantage of high-quality shots to score directly, and the trailing side often scores directly by fighting. Accordingly, more high-quality shots transpire at the end of the game, and the proportion of poor shots decline. The percentage of particularly good and good shots increased by 1% (from 29 to 30%), poor and neutral shots increased 4% (from 45 to 43%). That is, numerous shots go from neutral and poor to good and extremely good. At the decisive stage of every set, high-level players are better at seizing every opportunity to make better shots. A previous study has presented similar conclusions and argued that elite table tennis players could turn in excellent performances in the ending period (Zhao, 2016). The results suggest that athletes should ensure the quality of every stroke and reduce unnecessary mistakes in the ending periods of the competition.

The present study has some limitations. Although we clarified the stroke differences amongst the initial, intermediate, and ending periods of each set, we did not analyze the ending periods of the last set separately. Although serving is also a part of shots in this sport, its unique characteristics prompted us to separate it from the types of strokes. For the particularity of the serve, subsequent studies may focus on the differences of serve in different periods.

## Conclusion

At different periods during a table tennis game, the strokes show different characters. The ending period shows significant differences from the initial periods in all dimensions: stroke position, stroke type, stroke placement and stroke efficacy. At the ending period, the use of chop type is reduced, the landing area transfers from the middle long to forehand short and more good shots are performed. At the end of every set, the players adopted a safe stroke position, performed offensive stroke types and deployed flexible stroke placement. To ensure the quality of the stroke, the player may become very conservative at the ending periods. Relaxing and breaking the routine may increase the likelihood of winning. Our findings suggest that athletes should still focus on the topspin stroke in daily training and pay attention to do exercises to cope with changes in placement in special situation, the coaches should pay special attention to the athletes’ psychology training and can construct the corresponding scenarios according to different match periods for players during their training. At the ending periods of a table tennis match, quality shots and smooth transitions are the key to winning.

## Data Availability Statement

The raw data supporting the conclusions of this article are available from the corresponding author by request, without undue reservation.

## Ethics Statement

This study was approved by the College of Physical Education and Sports Training Academic Committee, Shanghai University of Sport.

## Author Contributions

JW completed all the work independently and consented to the publication of the manuscript.

## Conflict of Interest

The author declares that the research was conducted in the absence of any commercial or financial relationships that could be construed as a potential conflict of interest.

## Publisher’s Note

All claims expressed in this article are solely those of the authors and do not necessarily represent those of their affiliated organizations, or those of the publisher, the editors and the reviewers. Any product that may be evaluated in this article, or claim that may be made by its manufacturer, is not guaranteed or endorsed by the publisher.
